# Gut dysbiosis and bacterial translocation in the aneurysmal wall and blood in patients with abdominal aortic aneurysm

**DOI:** 10.1371/journal.pone.0278995

**Published:** 2022-12-14

**Authors:** Ken Nakayama, Tadashi Furuyama, Yutaka Matsubara, Koichi Morisaki, Toshihiro Onohara, Tetsuo Ikeda, Tomoharu Yoshizumi

**Affiliations:** 1 Department of Surgery and Science, Graduate School of Medical Sciences, Kyushu University, Fukuoka, Japan; 2 Department of Vascular Surgery, National Hospital Organization Kyushu Medical Center, Fukuoka, Japan; 3 Department of Surgery and Endoscope Center, Oral Medicine Research Center, Fukuoka Dental College, Fukuoka, Japan; Hunan Agricultural University, CHINA

## Abstract

Inflammation plays a part in the development of abdominal aortic aneurysm (AAA), and the gut microbiota affects host inflammation by bacterial translocation. The relationship between abdominal aortic aneurysm and the gut microbiota remains unknown. This study aimed to detect bacterial translocation in the aneurysmal wall and blood of patients with abdominal aortic aneurysm, and to investigate the effect of the gut microbiota on abdominal aortic aneurysm. We investigated 30 patients with abdominal aortic aneurysm from 2017 to 2019. We analysed the aneurysmal wall and blood using highly sensitive reverse transcription-quantitative polymerase chain reaction, and the gut microbiota was investigated using next-generation sequencing. In the 30 patients, bacteria were detected by reverse transcription- quantitative polymerase chain reaction in 19 blood samples (detection rate, 63%) and in 11 aneurysmal wall samples (detection rate, 37%). In the gut microbiota analysis, the *Firmicutes*/*Bacteroidetes* ratio was increased. The neutrophil-lymphocyte ratio was higher (2.94 ± 1.77 vs 1.96 ± 0.61, P < 0.05) and the lymphocyte-monocyte ratio was lower (4.02 ± 1.25 vs 5.86 ± 1.38, P < 0.01) in the bacterial carrier group than in the bacterial non-carrier group in blood samples. The volume of intraluminal thrombus was significantly higher in the bacterial carrier group than in the bacterial non-carrier group in aneurysmal wall samples (64.0% vs 34.7%, P < 0.05). We confirmed gut dysbiosis and bacterial translocation to the blood and aneurysmal wall in patients with abdominal aortic aneurysm. There appears to be a relationship between the gut microbiota and abdominal aortic aneurysm.

## Introduction

Abdominal aortic aneurysm (AAA) is one of the most common aortic diseases, and the rupture of AAA is an important cause of death. AAAs are generally asymptomatic, and the mortality rate in patients with ruptured AAAs is approximately 75% [1]. The risk factors for AAA include smoking, male sex, age, and hypertension [2, 3]. There is only invasive treatment for AAA, such as open repair or endovascular repair. Medical treatments for AAA have not been developed. There are several causes of the development of AAA, such as atherosclerosis, and infectious and inflammatory diseases. Previous studies have suggested pathophysiological mechanisms of the development and progression of AAA, such as atherosclerosis, degeneration of connective tissue, an effect of inflammatory cells (e.g., lymphomonocytes and macrophages), and the role of matrix metalloproteinases [4]. However, an obvious mechanism of AAA remains unknown.

Inflammation may play a part in the development of AAA in animal models. Inflammatory cell types and markers have also been detected in human AAA [5]. An inflammatory reaction causes degeneration of collagen and elastic fibres of the aortic wall, which is an important characteristic of AAA lesions [4]. The gut microbiota affects host inflammation and the immune system [6]. Recent studies have suggested an association between the gut microbiota and various diseases, such as cardiovascular diseases [7, 8]. Bacterial translocation (BT) is an important aspect involved in the association of the gut microbiota with various diseases. BT is defined as the passage of viable bacteria from the gastrointestinal tract to extraintestinal sites, and BT can be the cause of sepsis and organ dysfunction [9]. Examples of BT include the detection of gut bacteria in blood of patients with type 2 diabetes, which is relevant to AAA, and its relation to insulin resistance, and the detection of bacteria in atherosclerotic lesions of patients with coronary heart disease [10, 11]. Although the relationship between AAA and the gut microbiota has previously been reported [12], there have been no reports on BT in patients with AAA.

This study aimed to detect BT in the aneurysmal wall and blood of patients with AAA using the highly sensitive reverse transcription-quantitative polymerase chain reaction (RT-qPCR) method, and to investigate the effect of the gut microbiota on AAA.

## Materials and methods

### Patients

In this study, performed a census survey conducted at Kyushu University Hospital between 2017 and 2019. Thirty patients with AAA who had open repair performed at our hospital and National Hospital Organization Kyushu Medical Center between May 2017 and April 2019 were enrolled in this study. The sample size was determined as 30 because approximately 30 patients with AAA undergo surgery each year in our hospital. All patients provided written informed consent prior to enrolment in the study. Patients who underwent surgery for ruptured AAA, impending ruptured AAA, or inflammatory AAA were excluded. We also excluded the patients who did not consent to inclusion in this study. The study protocol was approved by the institutional review board of Kyushu University (approval number: 29–74). This research complies with the Declaration of Helsinki.

### Samples

Samples of the aneurysmal wall, intraluminal thrombus, blood, and faecal material of patients were used. We carefully collected the former three samples to avoid contaminating them in the open aortic repair operation. A total of 3 g of the aneurysmal wall and intraluminal thrombus were additionally collected. Blood was washed from these samples by sterile saline and stored in RNAlater Stabilization Solution (Thermo Fisher Scientific, Waltham, MA, USA) in a −80°C freezer. A volume of 3 mL of blood was collected from an arterial line and stored in a −80°C freezer after adding 1 mL of blood to 2 mL of RNAprotect Bacteria Reagent (Qiagen, Hilden, Germany). Faecal samples were collected on the second day before the operation after admission and stored at −80°C until DNA extraction.

### RNA extraction from aneurysmal wall, thrombus, and blood samples

After adding the RNAprotect Bacteria Reagent nine times, aneurysmal wall and thrombus samples were homogenised and suspended. Blood samples stored in the RNAprotect Bacteria Reagent were centrifuged (14000 × g, 10 min), and the pellet was used for RNA extraction. RNA extraction from aneurysmal wall, thrombus, and blood samples was performed by modified methods as previously described [13, 14]. Briefly, RNA was isolated using a modified acidic guanidinium thiocyanate-phenol-chloroform extraction method. Samples were resuspended with 346.5 μL of RLT lysis buffer (Qiagen, Hilden, Germany), 3.5 μL of β-mercaptoethanol, and 100 μL of Tris-EDTA buffer, and 300 mg of glass beads (diameter, 0.1 mm) were added to the suspension. The mixture was then vortexed for 5 min using a FastPrep FP 120 (MP Biomedicals, Irvine, CA, USA) at a power level of 5.0, and 500 μL of acid phenol was added and the mixture was incubated. After 100 μL of chloroform-isoamyl alcohol was added and centrifugated, the supernatant was collected and subjected to isopropanol precipitation. Finally, the nucleic acid fraction was suspended in nuclease-free water.

### RT-qPCR

The detection of bacteria was performed using the sensitive bacterial ribosomal RNA (rRNA)-targeted RT-qPCR method as previously described [13]. The RT-qPCR analysis was conducted using a Qiagen OneStep RT-PCR kit. Group- or species-specific primers were used for RT-qPCR. Twenty-two types of target bacteria are shown in the supporting information online (S1 Table). These bacteria cover ≥ 70% of the entire bacterial populations in healthy adults’ faeces. The results of RT-qPCR were assessed by a calibration curve, which was obtained from the corresponding number of bacteria. A standard curve was generated with the RT-qPCR data using the threshold cycle value for dilution series of the reference strains. Threshold cycle values of RNA extracted from the samples were applied to the standard curve to obtain the corresponding number of bacterial cells in a sample.

### DNA extraction from faecal samples

DNA extraction from faecal samples was performed using methods described previously with slight modifications [13]. Briefly, faecal samples were suspended to 10 times with phosphate-buffered saline, and 200 μL of the suspensions were re-suspended in 250 μL of extraction buffer (200 mM Tris-HCl, 80 mM EDTA, pH 9.0) and 50 μL of 10% sodium dodecyl sulphate. A total of 300 mg of glass beads (diameter, 0.1 mm) and 500 μL of Tris-EDTA buffer-saturated phenol (Nacalai Tesque, Inc., Kyoto, Japan) were added to the suspension, and the mixture was vortexed for 60 s by using a FastPrep FP 120 (MP Biomedicals, Irvine, CA, USA) at a power level of 5.0. After centrifugation (20380 × g for 5 min), 400 μL of the supernatant was collected. Phenol–chloroform extractions were performed, and 250 μL of the supernatant was subjected to isopropanol precipitation. Finally, the DNA was suspended in 1 mL of TE buffer (10 mM Tris–HCl, 1 mM EDTA, pH 8.0).

### Gut microbiota analysis

The V3–V4 region of the 16S rRNA gene was amplified by PCR, and amplicons were sequenced using Miseq (Illumina, Inc., San Diego, CA, USA), as previously described [15]. Sequencing data were then processed, and diversity trends were analysed using QIIME (www.qiime.org). Microbial α-diversity was evaluated by calculating the Shannon index and Chao1. Alpha diversity is described in terms of the richness or evenness. The Shannon index shows both richness and evenness of the species. Chao1 assesses the number of species in a community and represents richness. Gut dysbiosis was assessed by the ratio of the phyla *Firmicutes/Bacteroidetes* (*F/B*).

### Data analysis

We measured the neutrophil-lymphocyte ratio (NLR) and the lymphocyte-monocyte ratio (LMR) as indicators of inflammation from the peripheral blood cell count 2 days before the operation. The NLR and LMR were calculated from the absolute value of blood neutrophils, lymphocytes, and monocytes. The intraluminal thrombus thickness was determined at the point of maximal thickness. Which was measured in a slice of a preoperative contrast computed tomography (CT) scan, with the maximal aneurysmal diameter determined by axial imaging. The intraluminal thrombus volume was obtained by the ratio of intraluminal thrombus and the aneurysmal lumen at the same slice of contrast CT scan imaging.

### Statistical analysis

Categorical variables were assessed using Fisher’s exact test. Continuous variables were assessed using Student’s t-test, the paired t-test, or the Mann–Whitney U-test. A P value of < 0.05 was considered statistically significant. Statistical analysis was performed using the JMP software program, version 14.0 (SAS Institute, Inc., Cary, NC, USA).

## Results

### Characteristics of the patients

Sixty-nine surgical aortic repairs were performed, and 30 (43%) patients were included in this study. Tables 1 and 2 show the baseline characteristics of these patients. The median age of the patients was 66.9 years (range, 44–88 years), and most (93%) of the patients were men and had a smoking history (93%). We found that 10% of patients took intestinal drugs. No patients had antibiotics preoperatively.

**Table 1:** Baseline characteristics of the patients.

No.	Age (y)	Sex	BMI ^c^ (kg/m^2^)	Smoking	Hypertension	Diabetes	Aneurysm location	Aneurysm diameter (mm)	Aneurysm form	Thrombus thickness (mm)	Intraluminal thrombus volume (%)	PPI ^d^/H2 blocker^e^	Statin	Intestinal drug
1	65	M^a^	24.5	(+)	(+)	(-)	Infrarenal	47	Fusiform	13	37	(-)	(-)	(-)
2	62	M	17.1	(+)	(-)	(-)	Infrarenal	53	Fusiform	10	47	(-)	(+)	(-)
3	71	M	23.5	(+)	(-)	(-)	Infrarenal	45	Fusiform	25	26	(-)	(-)	(-)
4	64	M	33.1	(+)	(+)	(+)	Infrarenal	48	Fusiform	7	24	(-)	(+)	(-)
5	68	M	25.9	(+)	(-)	(+)	Infrarenal	53	Saccular	18	73	(-)	(+)	(-)
6	74	M	22.7	(+)	(-)	(-)	Infrarenal	52	Fusiform	2	15	(-)	(+)	(-)
7	72	M	25.9	(+)	(+)	(+)	Infrarenal	43	Fusiform	25	80	(+)	(+)	(-)
8	76	M	21.7	(+)	(-)	(-)	Infrarenal	58	Fusiform	22	75	(-)	(-)	(-)
9	75	M	21.3	(+)	(+)	(-)	Infrarenal	50	Fusiform	11	82	(+)	(-)	(-)
10	48	M	37.3	(+)	(+)	(-)	Common iliac	38	Fusiform	12	64	(-)	(+)	(+)
11	74	M	25.8	(+)	(+)	(-)	Infrarenal	51	Fusiform	26	77	(-)	(+)	(-)
12	63	M	23.1	(+)	(+)	(+)	Infrarenal	48	Fusiform	2	14	(-)	(+)	(-)
13	76	M	21.1	(+)	(-)	(+)	Infrarenal	53	Fusiform	20	76	(+)	(-)	(+)
14	64	M	16.0	(+)	(+)	(+)	Infrarenal	85	Fusiform	-	-	(+)	(-)	(-)
15	60	M	27.8	(+)	(-)	(-)	Infrarenal	43	Fusiform	0	0	(-)	(-)	(-)
16	70	M	25.3	(+)	(+)	(-)	Infrarenal	53	Fusiform	4	8	(-)	(+)	(-)
17	56	M	29.8	(+)	(+)	(-)	Infrarenal	80	Fusiform	28	55	(-)	(-)	(-)
18	69	F^b^	24.8	(+)	(+)	(-)	Infrarenal	35	Sacciform	5	24	(-)	(-)	(-)
19	77	F	24.4	(+)	(+)	(-)	Infrarenal	51	Fusiform	0	0	(-)	(+)	(-)
20	73	M	24.5	(+)	(-)	(-)	Infrarenal	49	Fusiform	4	20	(-)	(-)	(-)
21	64	M	21.5	(+)	(+)	(-)	Infrarenal	67	Fusiform	15	57	(-)	(+)	(-)
22	68	M	24.5	(+)	(+)	(-)	Infrarenal	53	Fusiform	23	60	(-)	(+)	(-)
23	73	M	25.5	(-)	(+)	(-)	Infrarenal	53	Fusiform	7	14	(-)	(-)	(-)
24	62	M	17	(+)	(-)	(-)	Infrarenal	60	Fusiform	15	35	(-)	(-)	(-)
25	66	M	23.4	(+)	(+)	(-)	Infrarenal	50	Fusiform	9	23	(+)	(+)	(-)
26	67	M	23.9	(+)	(-)	(-)	Infrarenal	50	Fusiform	26	71	(-)	(+)	(+)
27	77	M	25.2	(+)	(-)	(-)	Infrarenal	45	Fusiform	10	30	(+)	(+)	(-)
28	68	M	27.8	(+)	(+)	(+)	Infrarenal	54	Fusiform	25	73	(+)	(+)	(-)
29	68	M	24.8	(+)	(+)	(-)	Infrarenal	47	Fusiform	7	27	(+)	(+)	(-)
30	65	M	22.5	(+)	(+)	(-)	Infrarenal	50	Fusiform	15	49	(+)	(+)	(-)

^a^M; male.

^b^F; female.

^c^BMI; body mass index.

^d^PPI; proton pomp inhibitor.

^e^H2 blocker; histamine H2-receptor antagonist.

**Table 2:** Summary of the baseline characteristics of the patients.

Variable	n = 30
Age (y)	66.9±8.9
Male sex	28 (93%)
BMI^a^ (kg/m^2^)	24.2±4.2
Smoking	28 (93%)
Hypertension	18 (60%)
Diabetes	8 (27%)
Aneurysm diameter (mm)	52.1±10.3
Thrombus thickness (mm)	13.3±8.7
Intraluminal thrombus volume (%)	42.6±26.1
Medication	
PPI^b^/H2 blocker^c^	9 (30%)
Statin	18 (60%)
Intestinal drug	3 (10%)

Data are presented as the mean ± standard deviation or number (%).

^a^BMI; body mass index.

^b^PPI; proton pomp inhibitor.

^c^H2 blocker; histamine H2-receptor antagonist.

### Gut microbiota analysis

**Fig 1:**
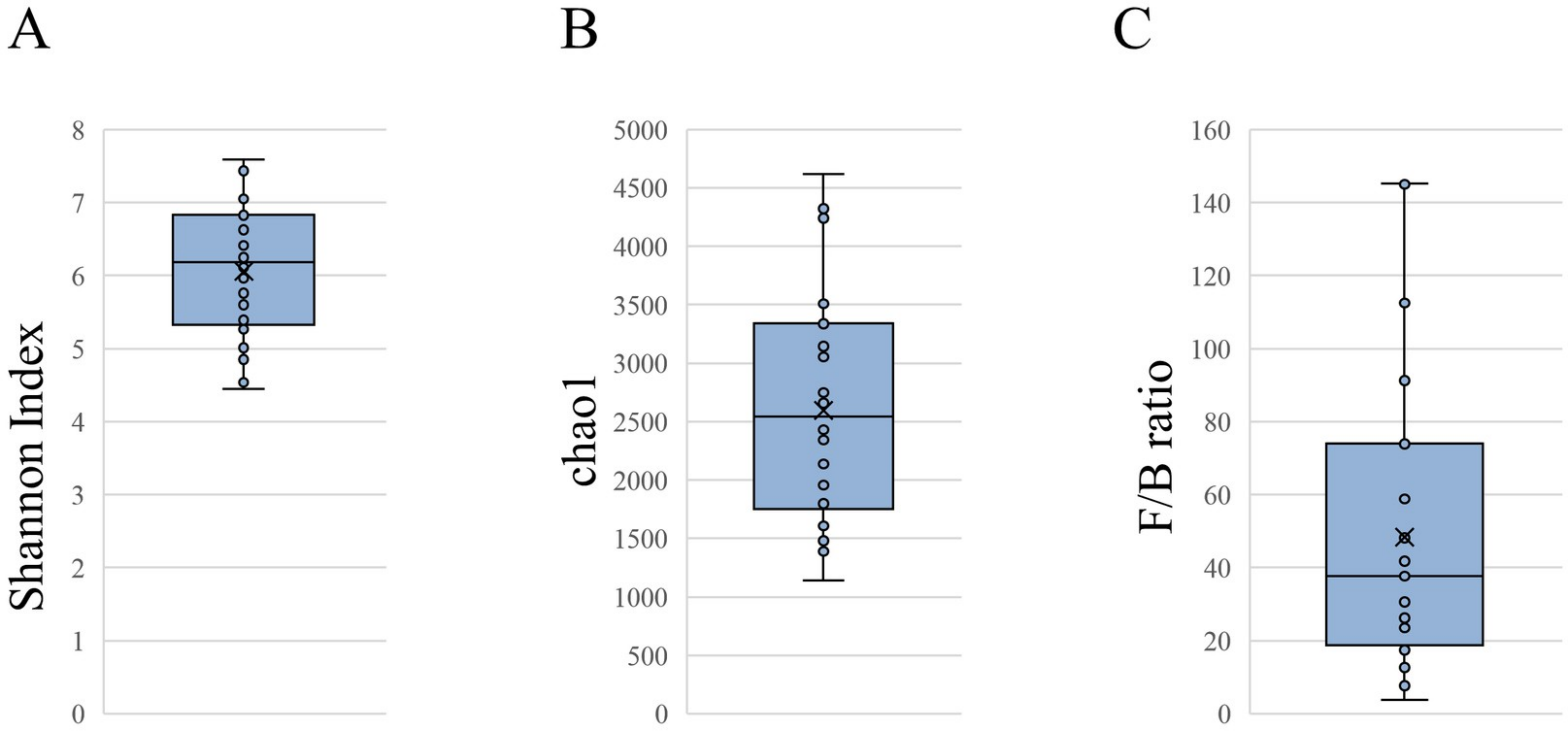
Gut microbiota analysis. Alpha diversity of the (A) Shannon index and (B) Chao1 in patients with abdominal aortic aneurysms. (C) Ratio of the phyla *Firmicutes/Bacteroidetes* (*F/B*).

We analysed the gut microbiota from faecal samples of the patients. The relative abundance of the phyla is shown in S1 Fig. The median of the Shannon index and Chao1 of all patients were 6.2 (range: 4.5–7.6; Fig 1A) and 2545 (range: 1143–4617; Fig 1B), respectively. The median abundance of the phylum *Bacteroidetes* abundance was 3.0% and the *F*/*B* ratio was 39.7 (Fig 1C), which indicated that the gut microbiota of patients with AAA was disturbed.

### Detection of bacteria by RT-qPCR

We collected aneurysmal wall and blood samples from all 30 participants. Thrombus samples were assessed in eight patients. In 19 blood samples (detection rate, 63%) and in 11 aneurysmal wall samples (detection rate, 37%), bacteria were detected by RT-qPCR (Tables 3 and 4). No bacteria were detected in the eight intraluminal thrombus samples. We assessed 22 primers of bacteria (S1 Table). Eight types of bacteria were detected from 19 blood samples (Table 3). The most common type of bacteria was *Streptococcus*, which was detected in eight blood samples (Table 3). No *Enterococcus* species was detected in blood samples (Table 3). Eleven types of bacteria were detected from 11 aneurysmal wall samples (Table 4). The most common type of bacteria was *Staphylococcus*, which was detected in seven aneurysmal wall samples (Table 4). *Enterobacteriaceae* was detected in four aneurysmal wall samples (Table 4). Bacteria were detected from blood and aneurysmal wall samples in eight patients. Among these eight patients, the types of bacteria were different between blood and the aortic wall in six patients. These findings indicated the diversity of BT and an association between the gut microbiota and aneurysmal wall.

**Table 3:** Results of RT-qPCR-positive blood samples.

	Sample (cells/mL)																		
Target bacteria	2	4	5	6	8	11	13	14	15	16	17	20	21	22	23	24	25	28	30
***Clostridium coccoides* group**	**-**	**-**	**-**	**2**	**-**	**-**	**-**	**-**	**-**	**-**	**-**	**-**	**-**	**-**	**-**	**-**	**-**	**-**	**-**
***Clostridium leptum* subgroup**	**-**	**-**	**-**	**7**	**-**	**-**	**-**	**-**	**-**	**-**	**-**	**-**	**-**	**-**	**-**	**-**	**-**	**-**	**-**
***Lactobacillus gasseri* subgroup**	**-**	**1**	**-**	**1**	**-**	**-**	**-**	**-**	**-**	**-**	**-**	**-**	**3**	**-**	**-**	**-**	**-**	**-**	**-**
***Lactobacillus casei* subgroup**	**4**	**-**	**-**	**-**	**16**	**-**	**-**	**-**	**-**	**-**	**4**	**-**	**-**	**-**	**16**	**-**	**-**	**-**	**-**
***Atopobium* cluster**	**-**	**-**	**-**	**2**	**-**	**-**	**44**	**28**	**-**	**-**	**-**	**-**	**-**	**-**	**-**	**-**	**-**	**-**	**-**
***Staphylococcus***	**-**	**22**	**26**	**46**	**-**	**-**	**-**	**-**	**65**	**19**	**-**	**-**	**-**	**-**	**28**	**-**	**-**	**-**	**-**
***Streptococcus***	**-**	**3**	**5**	**11**	**-**	**-**	**-**	**-**	**-**	**-**	**10**	**-**	**-**	**3**	**-**	**-**	**6**	**5**	**5**
***Clostridium perfringens***	**-**	**-**	**-**	**-**	**-**	**1**	**-**	**-**	**1**	**-**	**-**	**2**	**-**	**-**	**-**	**1**	**-**	**-**	**-**

**Table 4:** Results of RT-qPCR-positive aneurysmal wall samples.

	Sample (cells/g)										
Target bacteria	8	10	11	21	22	24	25	26	28	29	30
***Clostridium coccoides* group**	**1×10** ^**3**^	**-**	**-**	**2×10** ^**2**^	**-**	**-**	**-**	**-**	**-**	**-**	**-**
***Clostridium leptum* subgroup**	**3×10** ^**3**^	**-**	**-**	**-**	**-**	**-**	**-**	**-**	**-**	**-**	**-**
***Bifidobacterium***	**5×10** ^**3**^	**-**	**-**	**-**	**-**	**-**	**-**	**-**	**-**	**-**	**-**
***Lactobacillus gasseri* subgroup**	**1×10** ^**3**^	**-**	**-**	**-**	**-**	**-**	**-**	**1×10** ^**2**^	**-**	**-**	**-**
***Lactobacillus ruminis* subgroup**	**1×10** ^**3**^	**-**	**-**	**-**	**-**	**-**	**-**	**-**	**-**	**-**	**-**
***Atopobium* cluster**	**1×10** ^**4**^	**-**	**-**	**-**	**-**	**-**	**-**	**-**	**-**	**-**	**-**
***Prevotella***	**-**	**-**	**-**	**-**	**-**	**-**	**-**	**-**	**-**	**4×10** ^**2**^	**-**
***Enterobacteriaceae***	**-**	**-**	**-**	**4×10** ^**2**^	**6×10** ^**2**^	**9×10** ^**2**^	**4×10** ^**2**^	**-**	**-**	**-**	**-**
***Staphylococcus***	**-**	**4×10** ^**2**^	**2×10** ^**2**^	**-**	**-**	**2×10** ^**2**^	**1×10** ^**2**^	**-**	**1×10** ^**3**^	**6×10** ^**2**^	**1×10** ^**3**^
***Streptococcus***	**-**	**-**	**-**	**-**	**-**	**-**	**4×10** ^**2**^	**-**	**-**	**50**	**-**
***Clostridium perfringens***	**-**	**-**	**-**	**-**	**-**	**1×10** ^**2**^	**-**	**-**	**2×10** ^**2**^	**-**	**-**

### Comparison of characteristics of the patients with or without bacteria

We next examined the association between bacterial carriage and the patients’ characteristics (Tables 5 and 6). In patients who had bacteria detected in blood, there were significantly higher neutrophil (P = 0.03) and monocyte counts (P = 0.02, Table 5), but a lower lymphocyte count, in the bacterial carrier group than in the bacterial non-carrier group (P = 0.04, Table 5). Consequently, in blood, the NLR was higher (P = 0.04, Table 5) and the LMR was lower (P<0.01, Table 5) in the bacterial carrier group than in the bacterial non-carrier group. In patients who had bacteria detected in the aneurysmal wall, there was a significantly higher volume of intraluminal thrombus in the bacterial carrier group than in the bacterial non-carrier group (P = 0.04, Table 6). Additionally, in these patients, the median intraluminal thrombus volume in the bacterial carrier group was 64.0% (range, 6%–77%) and that in the bacterial non-carrier group was 34.7% (range, 0%–76%).

**Table 5:** Comparison of the characteristics between bacterial non-carriers and carriers in blood.

Variable	Bacterial non-carrier in blood (n = 11)	Bacterial carrier in blood (n = 19)	P value
**Age (y)**	**68.4±8.2**	**67.5±5.7**	**0.74**
**Male sex**	**9 (82)**	**19 (100)**	**0.13**
**BMI**^a^ **(kg/m**^**2**^**)**	**25.3±4.2**	**23.8±4.4**	**0.37**
**Smoking**	**11 (100)**	**18 (95)**	**1**
**Hypertension**	**8 (73)**	**11 (58)**	**0.47**
**Diabetes**	**2 (18)**	**5 (26)**	**1**
**Aneurysm diameter (mm)**	**45.4±5.0**	**56.1±10.6**	**0.004**
**Thrombus thickness (mm)**	**12.4±9.3**	**13.9±8.9**	**0.67**
**Intraluminal thrombus volume (%)**	**33.8±25.3**	**40.2±23.8**	**0.50**
**Medication**			
**PPI**^b^**/H2 blocker**^c^	**4 (36)**	**5 (26)**	**0.69**
**Statin**	**7 (64)**	**11 (58)**	**1**
**Intestinal drug**	**2 (18)**	**1 (5)**	**0.54**
**Differential count of leukocytes**			
**Neutrophils (%)**	**56.8±6.4**	**63.3±9.4**	**0.03**
**Lymphocytes (%)**	**30.6±6.0**	**25.2±7.4**	**0.04**
**Monocytes (%)**	**5.3±0.9**	**6.4±1.5**	**0.02**
**NLR** ^d^	**1.96±0.61**	**2.94±1.77**	**0.04**
**LMR** ^e^	**5.86±1.38**	**4.02±1.25**	**<0.01**

Data are presented as the mean ± standard error or number (%).

^a^BMI; body mass index.

^b^PPI; proton pomp inhibitor.

^c^H2 blocker; histamine H2-receptor antagonist.

^d^NLR; neutrophil-to-lymphocyte ratio.

^e^LMR; lymphocyte-to-monocyte ratio.

**Table 6:** Comparison of the characteristics between bacterial non-carriers and carriers in aneurysmal wall.

Variable	Bacterial non-carrier in the aneurysmal wall (n = 19)	Bacterial carrier in the aneurysmal wall (n = 11)	P value
**Age (y)**	**68.9±6.2**	**66.0±7.2**	**0.26**
**Male sex**	**17 (90)**	**11 (100)**	**0.52**
**BMI**^a^ **(kg/m**^**2**^**)**	**24.3±3.9**	**24.6±5.1**	**0.87**
**Smoking**	**18 (95)**	**11 (100)**	**1**
**Hypertension**	**11 (58)**	**8 (73)**	**0.47**
**Diabetes**	**6 (32)**	**1 (9)**	**0.22**
**Aneurysm diameter (mm)**	**51.9±11.8**	**52.5±7.5**	**0.87**
**Thrombus thickness (mm)**	**9.9±8.2**	**15.5±7.4**	**0.07**
**Intraluminal thrombus volume (%)**	**34.7±2.5**	**64.0±9.6**	**0.04**
**Medication**			
**PPI**^b^**/H2 blocker**^c^	**5 (26)**	**4 (36)**	**0.69**
**Statin**	**9 (47)**	**9 (82)**	**0.12**
**Intestinal drug**	**1 (5)**	**2 (18)**	**0.54**
**Differential count of leukocytes**			
**Neutrophils (%)**	**60.9±9.1**	**61.0±8.9**	**0.96**
**Lymphocytes (%)**	**26.5±7.1**	**28.3±7.8**	**0.54**
**Monocytes (%)**	**6.0±1.4**	**6.1±1.5**	**0.82**
**NLR** ^d^	**2.67±1.74**	**2.42±1.10**	**0.67**
**LMR** ^e^	**4.65±1.71**	**4.77±1.34**	**0.83**

Data are presented as the mean ± standard error or number (%).

^a^BMI; body mass index.

^b^PPI; proton pomp inhibitor.

^c^H2 blocker; histamine H2-receptor antagonist.

^d^NLR; neutrophil-to-lymphocyte ratio.

^e^LMR; lymphocyte-to-monocyte ratio.

## Discussion

In this study, we found gut dysbiosis in patients with AAA, with a decrease in the abundance of the phylum *Bacteroidetes* and an increase in the *F*/*B* ratio. Other previous reports evaluated the gut microbiota in patients with atherosclerotic diseases as follows [16–18]. Emoto et al. reported that the *F*/*B* ratio was higher in patients with coronary artery disease (*F*/*B* ratio: 1.6 ± 1.0) than in age- and sex-matched controls with no coronary artery disease (*F*/*B* ratio: 1.3 ± 2.0) and healthy volunteers (*F*/*B* ratio: 1.1 ± 1.4) [16]. Szabo et al. showed that an increased *F*/*B* ratio was associated with an increased carotid intima–media thickness (mean *F*/*B* ratio of intima–media thickness > 0.9 vs intima–media thickness < 0.9 groups: 2.299 vs 1.436, P = 0.031) [17]. Additionally, gavage of some species of *Bacteroidetes* prevented the formation of atherosclerotic plaques [18]. Another study showed that mice with more atherosclerotic plaques showed a high *F*/*B* ratio [19]. Atherosclerosis appears to be related to the progression of AAA. Our results are consistent with these studies, and they suggest that an increase in the *F*/*B* ratio in the gut microbiota is an important aspect of development of atherosclerosis and AAA.

To the best of our knowledge, this is the first study to detect bacteria from the aneurysmal wall and blood using the RT-qPCR method and to confirm BT in patients with AAA. Our highly sensitive RT-qPCR method enabled the detection of the presence of bacteria in the AAA wall and in blood of patients with AAA. RT-qPCR used in this study followed the method for targeting rRNA molecules that was developed by Matsuda et al. [13] rRNA is a universal constituent of bacterial ribosomes and shows high copy numbers in a single bacterial cell. The rRNA-targeted RT-qPCR is 100- to 1000-fold more sensitive than conventional PCR targeting DNA. A study reported that, in blood samples of patients with neutropenia and fever, the bacterial detection rate (69.6%) by bacterial rRNA-targeted RT-PCR was higher than that by blood culture (17.4%, P < 0.001) [14]. This finding suggested the usefulness and reliability of this method. Moreover, Sato et al. reported that the detection rate of bacteria in blood samples was 28% (14/50) in patients with type 2 diabetes and 4% (2/50) in controls by a similar method [10]. The detection rates in our study were 63% in blood and 37% in the aneurysmal wall. Although 8 (28%) of 30 our patients had diabetes, our detection rates were remarkably high. This finding suggested an association between bacterial detection and AAA.

The types of bacteria detected from aneurysmal wall samples were the *Clostridium coccoides* group, *Clostridium leptum* subgroup, genus *Bifidobacterium*, *Lactobacillus gasseri* subgroup (*Lactobacillus*), *Lactobacillus ruminis* subgroup (*Liquorilactobacillus* and *Ligilactobacillus*), *Atopobium* cluster, genus *Prevotella*, genus *Enterobacteriaceae* (formerly taxonomic nomenclature), genus *Staphylococcus*, genus *Streptococcus*, and *Clostridium perfringens*. Detection of this wide range of typical bacteria as gut microbiota suggested that nonspecific bacteria translocated to blood or aneurysmal wall, and that commensal bacteria of the skin or oral cavity was unlikely to have contaminated the samples. The former five types of bacteria include intestinal resident bacteria and some of them are used as probiotics. There have been few clinical cases where these five bacteria caused adverse events, and only several reports that a relative reduction in them in the gut microbiota was related to developing inflammatory diseases and anti-inflammatory effects [20–24]. In contrast, the latter six types of bacteria, which are detected at a higher frequency than the former five types, are often pathogenic. Sato et al. reported that *Atopobium* cluster was detected at a significantly higher rate in patients with type 2 diabetes than in controls (detection rate: 14% vs 0%, P < 0.05) [10]. In our study, *Atopobium* cluster was detected from three blood samples of patients with AAA, and two of them had type 2 diabetes. The detection rate of *Atopobium* cluster in all eight patients with type 2 diabetes was 25%, which appeared to be a reasonable result compared with Sato et al.’s report [10]. The detection of *Atopobium* cluster from blood samples of patients with type 2 diabetes may be affected by that disease. In addition, an association between *Atopobium* and tuboovarian abscess or bacterial vaginosis has been suggested [25, 26]. *Prevotella* is related to inflammatory periodontal diseases [27], and *Enterobacteriaceae* causes enteric infection. *Staphylococcus* produces enterotoxin and is one of the most common types of bacteria that result in food poisoning [28]. *Streptococcus* is associated with tonsillitis and acute glomerulonephritis. *Clostridium perfringens* causes gas gangrene. These bacteria also cause the development of an inflammatory response in various sites *in vivo*. Regarding the progression of AAA, previous studies have shown that an inflammatory response in the arterial wall plays an important role [29].

In this study, we found bacteria in the blood and aneurysmal wall. Blood samples in the bacterial carrier group showed a high differential count of neutrophils and monocytes, high NLR, and low LMR. The NLR and LMR are objective parameters, which indicate an inflammatory response, and are predictors of various diseases [30–32]. In vascular disorders, the LMR is related to the severity of coronary artery disease [33]. Xie et al. [34] suggested that a low LMR indicates a greater inflammatory response in the aortic wall, and that patients with thoracic aortic aneurysms with a high LMR are more likely to have type I endoleak during thoracic endovascular aortic repair. The present study showed that there was an association between detected bacteria and inflammation in the blood. In addition, bacterial detection in the aneurysmal wall suggested that these bacteria affect the progression of AAA by involving inflammation in aneurysmal wall.

Although there have been many studies regarding the role of intraluminal thrombus in the progression and rupture of AAA, many details of this process are still unclear. The development of intraluminal thrombus is mainly caused from the activation of platelets associated with a turbulent and stagnant blood flow [35], and there is a potential benefit of antiplatelet treatment of medium-sized AAAs [36]. Haller et al. reported that intraluminal thrombus of AAA might be a marker of aortic wall weakening and it was associated with early rupture [37]. In our study, the intraluminal thrombus volume tended to be higher in the bacterial carrier group than in the bacterial non-carrier group in aneurysmal wall samples. However, the rate of antiplatelet medication was not different between the groups. The presence of bacteria in the aneurysmal wall may contribute to the progression of intraluminal thrombus and affect early rupture.

There are several limitations to this study that should be considered. First, the number of patients was small. Therefore, additional larger studies are required to verify our findings. Second, we could not obtain samples from patients without AAA because this was a census study of patients with AAA only. Non-AAA controls were not included in this study. Furthermore, collecting the aortic wall of patients without AAA was impossible because only patients with AAA underwent aortic surgery in the hospital and centre included in this study. Third, the primers for RT-qPCR in this study did not cover all bacterial strains. Accordingly, other bacteria not targeted in this study might be important. However, the types of primers used in our examination appear to be satisfactory.

## Conclusion

In conclusion, this study shows gut dysbiosis and bacterial translocation to the blood and aneurysmal wall in patients with AAA. Our findings suggest a relationship between the gut microbiota and AAA. The next step in this research protocol is to determine the localization of detected bacteria by using fluorescence in situ hybridization method. Additionally, further analyses are required to investigate the specific association of translocated bacteria and inflammation in the aneurysmal wall and blood. Furthermore, intervention of the gut microbiota may contribute to preventing the progression of AAA in the future.

## Supporting information

S1 Table Target bacteria and primers sequences. (DOCX)

S1 Fig Relative abundance of phyla in the gut microbiota. (DOCX)
