# Supplementary material for: Gut dysbiosis and bacterial translocation in the aneurysmal wall and blood in patients with abdominal aortic aneurysm
Source: PLoS One. 2022 Dec 14;17(12):e0278995. doi: 10.1371/journal.pone.0278995 (PMC9749999; doi:10.1371/journal.pone.0278995)
Supplement: S1 Table — (DOCX) [file pone.0278995.s001.docx]

**Supporting information**

**S1 Table. Target bacteria and primers sequences**

| Target bacteria | Primer | Sequence (5’-3’) | Reference |
| --- | --- | --- | --- |
| *Clostridium coccoides* group | g-Ccoc-F  g-Ccoc-R | AAATGACGGTACCTGACTAA  CTTTGAGTTTCATTCTTGCGAA | 1 |
| *Clostridium leptum* subgroup | sg-Clept-F  sg-Clept-R3 | GCACAAGCAGTGGAGT  CTTCCTCCGTTTTGTCAA | 2 |
| *Bacteroides fragilis* group | g-Bfra-F  g-Bfra-R | ATAGCCTTTCGAAAGRAAGAT  CCAGTATCAACTGCAATTTTA | 1 |
| *Bifidobacterium* | g-Bifid-F  g-Bifid-R | CTCCTGGAAACGGGTGG  GGTGTTCTTCCCGATATCTACA | 3 |
| *Atopobium* cluster | c-Atopo-F  c-Atopo-R | GGGTTGAGAGACCGACC  CGGRGCTTCTTCTGCAGG | 2 |
| *Prevotella* | g-Prevo-F  g-Prevo-R | CACRGTAAACGATGGATGCC  GGTCGGGTTGCAGACC | 1 |
| *Enterobacteriaceae*  (formerly taxonomic nomenclature) | En-lsu3F  En-lsu3’R | TGCCGTAACTTCGGGAGAAGGCA  TCAAGGCTCAATGTTCAGTGTC | 4 |
| *Enterococcus* | Ec-ssu1 F  Ec-ssu1R | GGATAACACTTGGAAACAGG  TCCTTGTTCTTCTCTAACAA | 5 |
| *Staphylococcus* | g-Staph-F  g-Staph-R | TTTGGGCTACACACGTGCTACAATGGACAA  AACAACTTTATGGGATTTGCWTGA | 5 |
| *Streptococcus* | F  R | GCTTAGAAGCAGCTATTCATTC  GGATACACCTTTCGGTCTCTC | 6 |
| *Clostridium perfringens* | s-Clper-F  ClPER-R | GGGGGTTTCAACACCTCC  GCAAGGGATGTCAAGTGT | 5, 7 |
| *Clostridioides (Clostridium) difficile* | Cd-lsu-F  Cd-lsu-R | GGGAGCTTCCCA TAC GGG TTG  TTG ACT GCC TCA ATGCTT GGG C | 8 |
| *Pseudomonas* | PSD7F  PSD7R | CAAAACTACTGAGCTAGAGTACG  TAAGATCTCAAGGATCCCAACGGCT | 4 |
| *Lactobacillus*  (*Lactobacillus gasseri* subgroup) | sg-Lgas-F  sg-Lgas-R | GATGCATAGCCGAGTTGAGAGACTGAT  TAAAGGCCAGTTACTACCTCTATCC | 5 |
| *Lacticaseibacillus*  (*Lactobacillus casei* subgroup) | sg-Lcas-F  sg-Lcas-R | ACCGCATGGTTCTTGGC  CCGACAACAGTTACTCTGCC | 5 |
| *Latilactobacillus*  (*Lactobacillus sakei* subgroup) | sg-Lsak-F  sg-Lsak-R | CATAAAACCTAMCACCGCATGG  TCAGTTACTATCAGATACRTTCTTCTC | 5 |
| *Liquorilactobacillus and Ligilactobacillus* (*Lactobacillus ruminis* subgroup) | sg-Lrum-F  sg-Lrum-R | CACCGAATGCTTGCAYTCACC  GCCGCGGGTCCATCCAAAA | 5 |
| *Limosilactobacillus except L. fermentum (Lactobacillus reuteri* subgroup) | sg-Lreu-F  sg-Lreu-R | GAACGCAYTGGCCCAA  TCCATTGTGGCCGATCAGT | 5 |
| *Lactiplantibacillus*  (*Lactobacillus plantarum* subgroup) | sg-Lpla-F  sg-Lpla-R | CTCTGGTATTGATTGGTGCTTGCAT  GTTCGCCACTCACTCAAATGTAAA | 5 |
| *Levilactobacillus*  (*Lactobacillus brevis*) | s-Lbre-F  s-Lbre-R | ATTTTGTTTGAAAGGTGGCTTCGG  ACCCTTGAACAGTTACTCTCAAAGG | 5 |
| *Limosilactobacillus*  (*Lactobacillus fermentum*) | LFer-1  LFer-2 | CCTGATTGATTTTGGTCGCCAAC  ACGTATGAACAGTTACTCTCATACGT | 5 |
| *Levilactobacillus*  (*Lactobacillus brevis*) | s-Lbre-F  s-Lbre-R | ATTTTGTTTGAAAGGTGGCTTCGG  ACCCTTGAACAGTTACTCTCAAAGG | 5 |
| *Fructilactobacillus*  *(Lactobacillus fructivorans)* | s-Lfru-F  s-Lfur-R | TGCGCCTAATGATAGTTGA  GATACCGTCGCGACGTGAG | 5 |

**References**

1. Matsuki T, Watanabe K, Fujimoto J, Miyamoto Y, Takada T, Matsumoto K, et al. Development of 16S rRNA-gene-targeted group-specific primers for the detection and identification of predominant bacteria in human feces. Appl. Environ. Microbiol. 2002;68: 5445–5451.

2. Matsuki T, Watanabe K, Fujimoto J, Takada T, Tanaka R. Use of 16S rRNA gene-targeted group-specific primers for real-time PCR analysis of predominant bacteria in human feces. Appl. Environ. Microbiol. 2004;70: 7220–7228.

3. Matsuki T, Watanabe K, Tanaka R, Oyaizu H. Rapid identification of human intestinal bifidobacteria by 16S rRNA-targeted species- and group-specific primers. FEMS Microbiol. Lett. 1998;167: 113–121.

4. Matsuda K, Tsuji H, Asahara T, Kado Y, Nomoto K. Sensitive quantitative detection of commensal bacteria by rRNA-targeted reverse transcription-PCR. Appl Environ Microbiol. 2007;73: 32–39.

5. Matsuda K, Tsuji H, Asahara T, Matsumoto K, Takada T, Nomoto K. Establishment of an analytical system for the human fecal microbiota, based on reverse transcription-quantitative PCR targeting of multicopy rRNA molecules. Appl. Environ. Microbiol. 2009;75: 1961–1969.

6. Sakaguchi S, Saito M, Tsuji H, Asahara T, Takata O, Fujimura J, et al. Bacterial rRNA-targeted reverse transcription-PCR used to identify pathogens responsible for fever with neutropenia. J Clin Microbiol. 2010;48: 1624–1628.

7. Kikuchi E, Miyamoto Y, Narushima S, Itoh K. Design of species-specific primers to identify 13 species of Clostridium harbored in human intestinal tracts. Microbiol. and Immunol. 2002;46: 353–358.

8. Matsuda K, Tsuji H, Asahara T, Takahashi T, Kubota H, Nagata S, et al. Sensitive quantification of Clostridium difficile cells by reverse transcription-quantitative PCR targeting rRNA molecules. Appl. Environ. Microbiol. 2012;78: 5111–5118.
