# Supplementary figures and images for: Gut dysbiosis and bacterial translocation in the aneurysmal wall and blood in patients with abdominal aortic aneurysm
Source: PLoS One. 2022 Dec 14;17(12):e0278995. doi: 10.1371/journal.pone.0278995 (PMC9749999; doi:10.1371/journal.pone.0278995)

**Supporting information**

**S1 Fig. Relative abundance of phyla in the gut microbiota.**


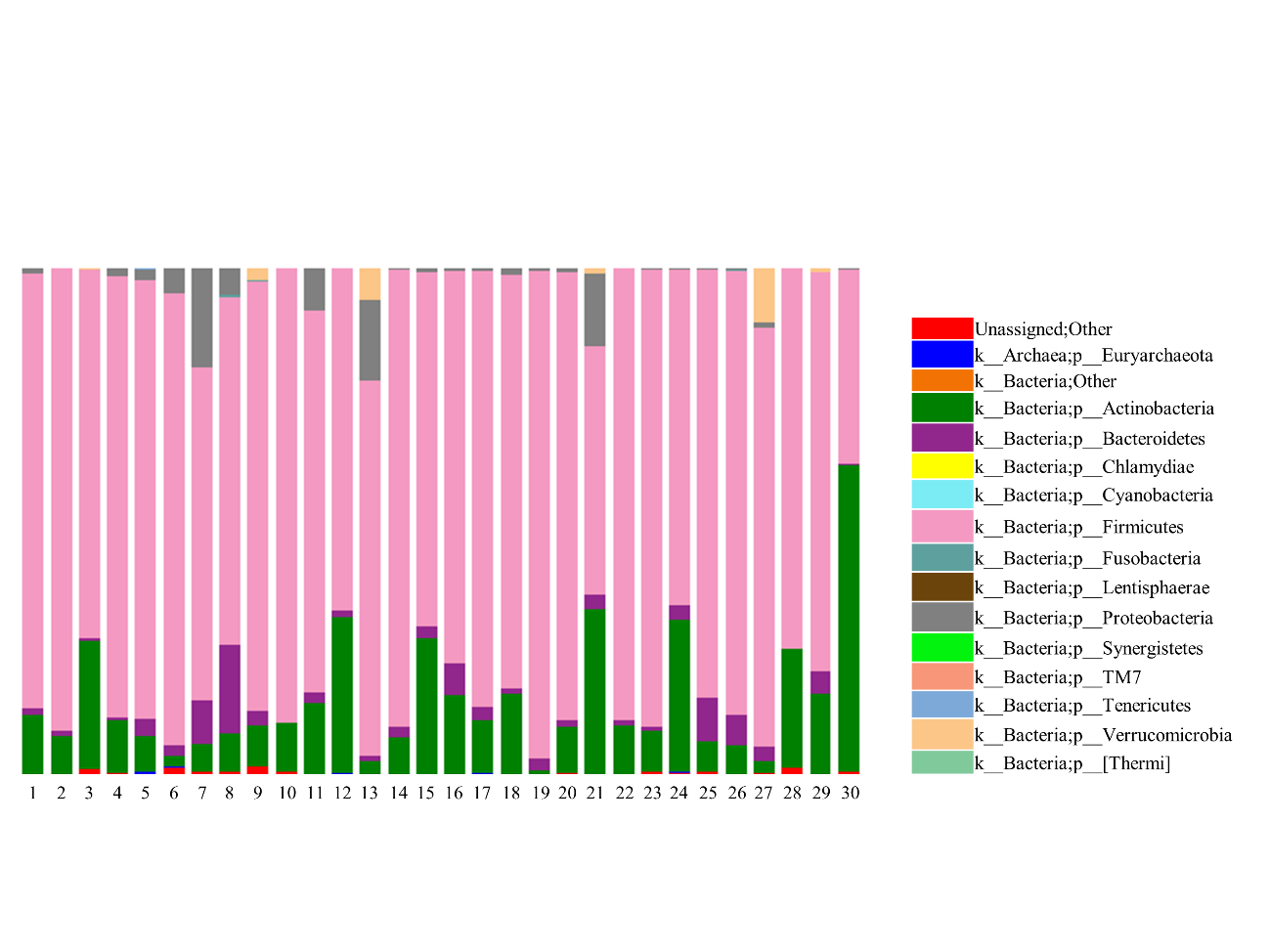

Supplement: S1 Fig — (DOCX) [file pone.0278995.s002.docx]
